# Robust Tract Skeleton Extraction of Cingulum Based on Active Contour Model from Diffusion Tensor MR Imaging

**DOI:** 10.1371/journal.pone.0056113

**Published:** 2013-02-26

**Authors:** Wu Li, Xiaoping Hu

**Affiliations:** 1 State Key Laboratory of Management and Control for Complex Systems, Institute of Automation, Chinese Academy of Sciences, Beijing, China; 2 Department of Biomedical Engineering, Georgia Tech and Emory University, Atlanta, Georgia, United States of America; Hangzhou Normal University, China

## Abstract

Cingulum is widely studied in healthy and psychiatric subjects. For cingulum analysis from diffusion tensor MR imaging, tractography and tract of interest method have been adopted for tract-based analysis. Because tractography performs fiber tracking according to local diffusion measures, they can be sensitive to noise and tracking errors can be accumulated along the fiber. For more accurate localization of cingulum, we attempt to define it by skeleton extraction using the tensors' information throughout the tract of cingulum simultaneously, which is quite different from the idea of tractography. In this study, we introduce an approach to extract the skeleton of cingulum using active contour model, which allows us to optimize the location of cingulum in a global sense based on the diffusion measurements along the entire tract and contour regularity. Validation of this method on synthetic and experimental data proved that our approach is able to reduce the influence of noise and partial volume effect, and extract the skeleton of cingulum robustly and reliably. Our proposed method provides an approach to localize cingulum robustly, which is a very important feature for tract-based analysis and can be of important practical utility.

## Introduction

With diffusion tensor magnetic resonance imaging (DT-MRI), diffusion anisotropy effects can be ascertained, characterized, and exploited to provide informative details regarding white matter microstructure [1]. DT-MRI makes it possible to noninvasively study three-dimensional geometric structure of specific fiber tracts [2] and possible micro-structural connectivity between different brain regions. To date, DT-MRI is widely used in basic neuroscience research and clinical applications [3]–[8].

Cingulum is part of the brain's limbic system, which is involved in humans' cognition, emotion, depression, motor function, etc. [5], [9], [10] It forms a single and continuous structure. Cingulum has been widely studied in many clinical researches by DT-MRI. Wang et al. [3] especially studied anterior cingulum abnormalities in male patients with schizophrenia; Catheline et al. [4] especially studied the alterations of the cingulum bundle during aging and Alzheimer's disease; Stenset et al. [5] studied the cingulum fiber diffusivity and CSF T-tau in patients with subjective and mild cognitive impairment; and many other researchers also focus on the diffusion analysis of cingulum.

For diffusion analysis of cingulum, region of interest (ROI) based method is usually adopted [3], [4], [5]. Interested ROI were manually defined on different parts of cingulum. As we all know, this method is laborious and operator dependent. And this approach limits a study to only being sensitive to changes in those few parts of the brain where ROIs are placed [11]. More sophisticated approach is tract of interest (TOI) analysis. In TOI, tractography is used to reconstruct the fiber bundles and then the diffusion values along/on the fiber tract are compared across subjects. For example, Zhang et al. [6] reconstructed cingulum tract by streamline tracking method [12] and analyzed fractional anisotropy (FA) in three parts of cingulum for disease analysis; Gong et al. [7] used tractography (one similar method to Lazar et al. [13]) to find cingulum bundle and FA was parameterized according to the position within the tract. TOI-based method makes it feasible for detailed diffusion analysis along/on the whole cingulum, which can provide more information for basic neuroscience research and clinical studies.

Fiber tracking is the most important step for TOI method and any deviation of tractography will lead to inaccurate results for following analysis. To ascertain fiber trajectory of cingulum from DT-MRI, various tractography methods could be used [14]. There are several widely used methods, including streamline tracking [12], [15]–[17] tensor deflection (TEND) tractography [13], [18], [19], and probabilistic diffusion tractography [20], [21]. These methods are based on integrating the local white matter orientation information in the DT-MRI data across the brain [22], [23]. Although they have been used successfully to track white matter structures of interest in various studies [22], [24], [25], they are sensitive to noise and tracking errors may cumulate along the fiber track [2], [17], [26]. Due to the influence of noise, partial volume effect (PVE), and fiber crossing/fanning/branching [15], [23], most existing tractography methods are known to miss fibers [21] or result in wrong pathways [8], [22]. To reduce the errors in tractography [23], knowledge-based multiple-ROI approaches have been adopted [7], [23], [27], [28]. Although these approaches impose a significant constraint on the tract to reduce the occurrence of erroneous results, they rely strongly on prior tractography results, so any limitations and sensitivities to the tractography algorithm may not be avoided [22].

Smith et al. [11] proposed the tract-based spatial statistics (TBSS) method for cross-subject analysis. This method gets the skeleton of the whole brain white matter tracts for following analysis. TBSS provides the idea of skeletonisation of whole brain fiber tracts. It is useful and effective for whole brain tract based analysis and is becoming more and more popular. Although TBSS can provide the skeleton for every subject by “back projection” [11], it firstly get the common skeleton from group subjects rather than the individual, and it provides the skeleton for whole brain white matter rather than one specific tract. Bringing the advantages of TOI and TBSS methods together, for more accurate localization of cingulum individually, we attempt to define it by skeleton extraction with all tensors' information throughout the tract based on active contour model.

The main segment of cingulum, which arching over corpus callosum, is studied in this paper. Here we describe a novel approach to extract the skeleton of cingulum individually, not based on tractography method but by searching the optimized skeleton based on active contour model and the tensors' information throughout this tract. Our purpose is to provide an optimal trajectory representation of cingulum by tract skeletonisation. The method determines the tract skeleton by global optimization, which can reduce the influence of noise and PVE and derive the orientation and shape of individual's cingulum pathway more robustly. Robustness and reliability, which is the most important requirements for the localization of cingulum, were tested on synthetic and real DT-MRI data.

## Methods

### Diffusion tensor MR data acquisition

DT-MRI data from six healthy subjects were used in this study. None of the subjects had (a history of) neurological or psychiatric disorders or anatomical abnormalities. This study was approved by the local medical ethical committee of Emory University. All participants gave written informed consent prior to study participation.

DT-MRI data were obtained on a 3.0 T MRI scanner (Siemens Medical Solutions, Malvern, PA) using diffusion weighted echo planar imaging with 12 different diffusion gradient directions (TR/TE: 6500/90 ms, matrix: 256×256, FOV: 220×220 mm, slice thickness 2.5 mm, b value: 1000 s/mm^2^). On each subject, DT-MRI scans were performed six times for subsequent averaging to get data sets with different signal-to-noise ratio (SNR) levels for further evaluation.

### Image preprocessing

Before calculating the diffusion tensors from DT-MR images, each data set was first re-sampled to spatially isotropic dataset, and preprocessed to remove skull and correct eddy-current-induced artifacts using FMRIB Software Library (FSL) tools (FMRIB, Oxford, U.K.; [29], [30]).

### Estimation of diffusion tensor and diffusion anisotropy from DT-MR images

Diffusion tensor and its principle values and their orientations were derived using a standard algorithm. Using eigenvalues/eigenvectors, different anisotropy measures were computed [1], [31], [32] to map tensor data onto scalars and to quantitatively estimate the diffusion anisotropy. Fractional anisotropy (FA) was calculated using
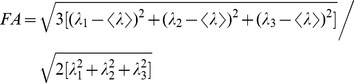
(1)

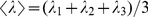
(2)where *λ*_1_, *λ*_2_, and *λ*_3_ are the eigenvalues of diffusion tensor *D*. The degree of anisotropy (tensor ellipsoid eccentricity) is related to the presence of oriented structures; and the main direction of diffusivities (main ellipsoid axes) is linked to the orientation in space of the structures [1]. Both the degree of anisotropy and tensor's eigenvectors provide important information of fiber's microstructure.

### Active contour based tract skeleton extraction

Our purpose is to provide an approach to extract the skeleton of cingulum accurately, not based on tractography but by searching the optimized skeleton based on active contour model. The main idea of our active contour based tract skeleton extraction (ACTSE) is to optimize the cingulum skeleton based on the tensors' information throughout the tract simultaneously, rather than trace the fiber path step by step and voxel by voxel based on local tensor information like tractography. So it is possible for ACTSE to reduce the influence of noise and PVE and derive the orientation and shape of individual's cingulum pathway more robustly.

In this study, the goal is to extract the skeleton of cingulum. Energy based active contour method (also named as “snake”) and curve fitting are adopted to for the optimization. The concept of snake introduced by Kass et al [33] has been successfully used in edge detection, object segmentation, and object tracking [33], [34], [35]. The snake model is based on an energy minimizing spline, with the energy depending on its shape and location within the image. For the skeleton of cingulum *C* in diffusion tensor domain, we define the evolving curve as
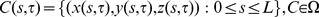
(3)where *L* denotes the length of the contour *C*, and *Ω* denotes the entire domain of brain mask. In Eq. (3), *τ* represents the evolution time; at each *τ*, there is one evolution of the contour. The continuous form in Eq. (3) can be approximated by a discrete representation as
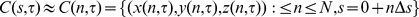
(4)where 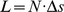, and 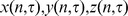 denote the 3D coordinates of point *n* at time 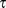. Here we set 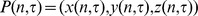. An energy function 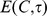 can be defined on the contour as [33]

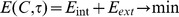
(5)where *E_int_* and *E_ext_*, respectively, represent the internal energy and external energy. The internal energy determines the regularity, and its minimization controls the smooth shape of the contour. Similar to other researchers [33], [34], [35, and more others], the definition for the internal energy is a quadratic functional given by
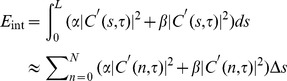
(6)where constants *α* and *β* are the weighting parameters that control the curve's tension and rigidity, and *C′* and *C″* denote the first and second derivatives of contour *C* with respect to *n*. Because curve fitting will be applied after curve evolution at each τ for more smoothed curve, *E_int_* is in fact eliminated in our study. That is, in our study *α* = 0, *β* = 0.

**Figure 1:**
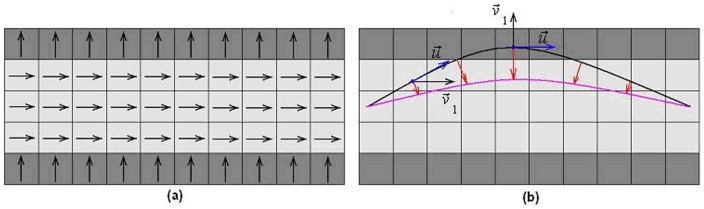
Illustration of contour evolution for ACTSE.

**Figure 2:**
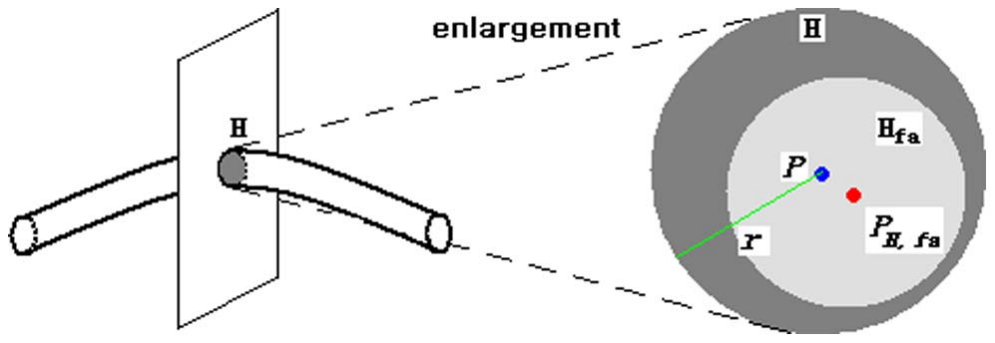
Illustration of parameters in the fourth item in *E_img_*. . Right round region is the enlargement of the dark region in Left image. *H* represents the common region of the tract's cross dissection at point *P(n,τ)* (blue point) along principle direction 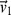 and the sphere with radius *r* (green line) at point *P(n,τ)*. *H_fa_* represents the region in *H* with *I_fa_*>0.1. *P_H,fa_(n,τ)* (red point) represents the center of *H_fa_*.

The external energy term determines the criteria of contour evolution dictated by FA value *I_fa_(x, y, z)* and eigenvectors, 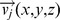 (*j* = 1,2,3), and is defined as
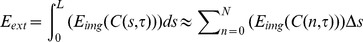
(7)where *E_img_(x,y,z)* denotes a scalar function; the local minimum of *E_img_* attracts the contour to an optimal location. Fig. 1 provides an illustration of the main idea for ACTSE. We select the function as a combination of a function of *FA* map's gradient and a function of eigenvector's changes.
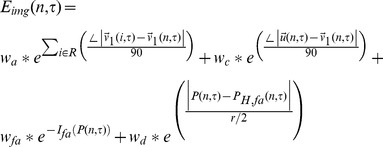
(8)There are four terms in *E_img_*. Constants *w_a_*, *w_c_*, *w_fa_* and *w_d_* are weighing factors for these terms. By experience, here we set *w_a_*, = 1, *w_c_* = 1, and *w_fa_* = 1. The first item defines the eigenvectors' consistency of contour's voxel with surrounding voxels. 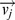 (*j* = 1,2,3) represent eigenvectors, 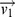 is the principle eigenvector. 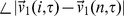 calculates the absolute angle difference between 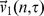 and 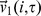. *R* is defined as the region surrounding voxel *n*, usually a 3×3×3 window. The first term keeps the tract skeleton away from the edge of the fiber bundle. The second term in *E_img_* defines the difference between contour's tangent vector 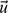 and the principle eigenvector. This term is included to make the tract skeleton's tangent as consistent as possible with voxel's principle vector. The third term in *E_img_* defines the influence of FA value *I_fa_*. Due to PVE, FA value decreases at the interface of gray matter and white matter. Term three tends to move the tract skeleton to the voxels with high FA value. The fourth term in *E_img_* is determined by the distance between the possible tract's center line and the estimated skeleton. The center line of cingulum is defined as a line passing through the center of the fiber tract and keeping the tract's trajectory. The fourth term is only used for fine adjustment. If the difference between 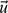 and 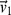 is larger than 30 degree, *w_d_* = 0 and if the iteration number is less than *N_s_*, *w_d_* = 0. Otherwise *w_d_* = 1. *N_s_* is set heuristically. At *N_s_*, there are no big changes for the evolution of tract skeleton. In our study, *N_s_* was set to 15. Maximum radius *r* of the tract is defined by the user to handle the possible situation where two different fiber bundles are crossing. *H* is defined as the overlapping area of the fiber tract's cross section at point *P(n,τ)* perpendicular to the principle direction 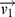 and the sphere with radius *r* at point *P(n,τ)*. *H_fa_* represents the region in *H* with *I_fa_*>0.1; all voxels in *H_fa_* should have the consistent principle eigenvector with point *P*(*n*,*τ*). *P_H,fa_(n,τ)* represents the center of *H_fa_*. The minimization of the fourth term aims to localize tract skeleton close to the centerline of the tract, as illustrated in Fig. 2. Overall, the minimization of *E_ext_* drives the skeleton to voxels with high *FA* and consistent eigenvectors and close to the center of fiber tract. The minimization of *E_ext_* controls the curve evolution of skeleton searching.

B-spline curve fitting is applied after curve evolution at each *τ* [22], further reducing the influence of noise and ensuring the smoothness of the skeleton. In addition, the path of contour *C* is represented by a three-dimensional cubic B-spline and re-parameterized by fixed distance along *C* at each *τ*. At each *τ*, energy 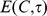 is minimized with the skeleton of cingulum 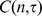 defined on the discretized grid. Subsequent curve fitting leads to a smoothed tract skeleton 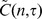 with subvoxel spatial resolution. The optimization of skeleton searching, including curve evolution and curve fitting, is performed iteratively. The distance between 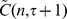 and 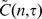, *D_s_*, is evaluated according to Eq. (9). The iteration stops when *D_s_* is less than a predefined constant 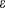.
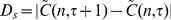
(9)


### Pseudocodes

To accurately localize the skeleton of cingulum in each individual, a series of consecutive curve evolution and curve fitting are performed iteratively. To begin the algorithm, a starting curve *C*(*n*,*0*) of cingulum's skeleton is initialized by mapping a reference curve to each individual according to the user-defined starting region, end region, and/or middle regions. Middle regions are selected based on possible cingulum's inflexion points. Reference curve is manually drawn based on the anatomical knowledge of cingulum. Based on the initial localization, we try to get the accurate skeleton of cingulum by the minimization of the defined energy function *E* and curve fitting iteratively with Eqs. (3), (4), (5), (7), (8) and (9).

The pseudocode for complete skeleton searching of cingulum is as follows:

**Begin**

Set *τ* = 0

Initialized localization of skeleton curve by mapping a reference one to each individual

Get *C* (*n*,0) (0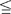 *n* 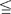 *N*)

Set *D_s_* = LARGE

While *D_s_*>
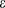


 For every voxel *n* at curve *C* (*n, τ*) (0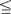 *n* 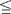 *N*)

  For every voxel *m* surrounding voxel *n*

   Calculate the energy *E* (*m*, *τ*)

   If *E* (*m*, *τ*)<*E* (*n*, *τ*),

    relocate point *n* to the updated location *m*

   End If

  Repeat for all *m* surrounding *n*

 Repeat for every voxel *n* at curve *C* (*n*, *τ*) (0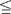 *n* 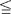 *N*)

 Calculate updated *C* (*n*, *τ+1*)

 Curve fitting to get a more smoothed tract skeleton 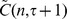


 Calculate *D_s_*

 Set *C* (*n*, *τ+1*) = 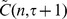


 Set *τ* = *τ*+1

End while

Output *C* (*n*, *τ*), the calculated skeleton of cingulum

**End**

### Evaluation of ACTSE method on synthetic data

**Figure 3:**
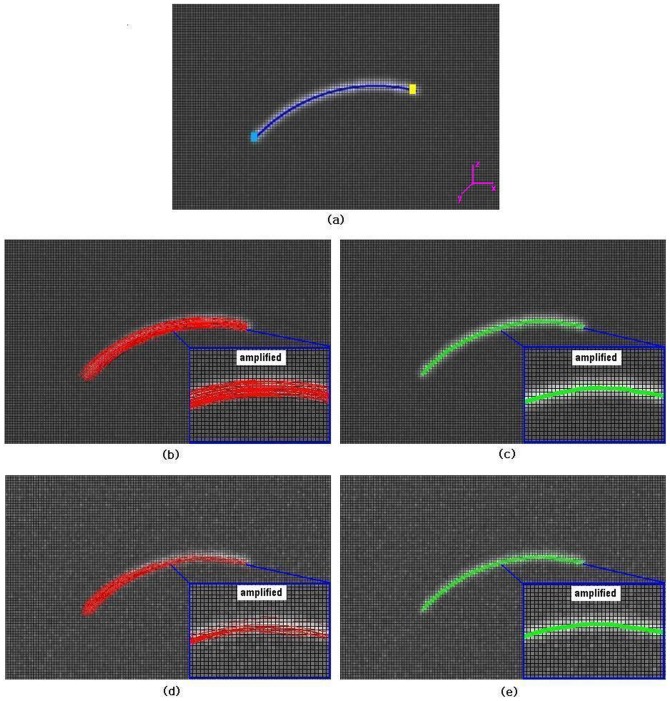
Illustration of extracted skeleton by ACTSE and tractography result by SLT on simulated phantom. (a) shows the ideal central line of simulated fiber tract overlaid on FA map (in blue). Light blue region and yellow region are defined as the starting and ending regions for the SLT. These regions also served as the constraints for ACTSE. (b) and (c) were reconstructed with added noise *sd = 0.003* (*SNR = 31.28*) and PVE level *t = 2*; (d) and (e) were reconstructed with noise *sd = 0.009* (*SNR = 10.67*) and *t = 2*. (b) and (d) show the fiber tractography results by SLT in red; (c) and (e) show the extracted tract skeleton by ACTSE in green. In (b), (c), (d) and (e), the blue rectangles amplified the same part of the results.

It is necessary to estimate the methods' robustness for a wide range of conditions to assess the performance of the method. Because there is no gold standard means for measuring fiber trajectories in living humans [17], [36], synthetic DT-MRI tensor data were generated. The data were simulated as a 3-D volume with 128×128×64 voxels, with one diffusion tensor *D* assumed for every voxel. Eigenvalues (*λ_1_*, *λ_2_*, *λ_3_*) and eigenvectors (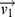,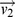,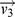) were assumed. One curved white matter tract structure was simulated and assumed to be an arc, as shown in Fig. 3(a). The voxels of simulated fiber tract were characterized by eigenvalues *λ_1_* = 1.0, *λ_2_* = *λ_3_* = 0.01, and the principle eigenvector 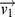 was assumed to have the same direction with fiber curve's tangent, the other two eigenvectors were set perpendicular to the principle one and to one another. For the voxels outside the simulated fiber tract, eigenvalues were set as *λ_1_* = *λ_2_* = *λ_3_* = 1 and the directions of eigenvectors were set randomly. Diffusion quantity, such as FA map, was calculated accordingly.

The simulated data was filtered by with the application of a 3×3×3 moving average different times (*t* = 1, 2, 3) to simulate different PVE levels. Moreover, in order to reflect the uncertainty in the estimation of the eigenvectors, random noise was added to the simulated diffusion tensors with different standard deviation (SD) (*sd* = 0.003, 0.006, 0.009, 0.012, 0.015) respectively, to generate different SNR levels. Here SNR is defined as
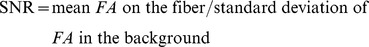



Both our ACTSE method and tractography could provide the geometric trajectory of cingulum. So here we compared the skeleton extracted by ACTSE with the center line acquired by the streamline tractography (SLT) method [12] on dataset with different PVE and noise levels. For the evaluation of synthetic data, the center line acquired by SLT is defined as the line connected by the continuous centroids of the cross sections along x-axial direction of all tracked fiber bundles. As shown in Fig. 3(a), it is easy to get the cross sections when sliced perpendicular to the x-axis. The deflection errors, defined as the distance along the arc length between the ideal center line of simulated curved tract (as shown in Fig. 3(a)) and the estimated tract skeleton by ACTSE or the calculated center line of tracked fiber tract by SLT, were quantitatively calculated. The mean error, between the location of the acquired tract skeleton by ACTSE or the calculated center line of tracked fiber bundles by SLT and the ideal tract center line, was also quantitatively calculated at different noise or PVE levels.

Starting and ending regions were defined as boxes placed manually at the two ends of the fiber trajectory, as shown in Fig. 3(a). For SLT, these regions served as seed regions and as stopping criteria. SLT was applied with step size = 1 voxel, curvature threshold = 30°. For SLT, in order to obtain as many fiber bundles as possible and obtain the tract as long as possible, no initial seed threshold or tracking FA threshold restriction were used; meanwhile a small curvature threshold was set to 30° to avoid erroneous tracking due to noise and PVE. ACTSE was applied with the starting and ending regions as two ends of the skeleton, and the step size of curve evolution was set to 1 voxel. The initial contour for skeleton searching was acquired by mapping the reference curve directly to the two ROIs. The reference curve is a roughly manually drawn curve line. The possible maximum radius of the simulated fiber *r* is set to 2 voxels.

### Evaluation of ACTSE on experimental data

ACTSE was applied on data from 6 healthy subjects. Each subject was scanned 6 times to acquire 6 datasets. For every subject, we randomly combined 5 acquisitions, 3 acquisitions, and 1 acquisition, respectively, resulting in 3 different SNR, each with 6 different data sets and these 6 data sets were independent with each other. This made it possible to estimate the deviation across 6 acquired scans at three noise levels respectively for every subject and estimate the average deviation of all subjects at same noise level.

SLT was only used for qualitative comparison. Seed regions and two end regions were manually defined on cingulum by expert for SLT. Color-coded FA map could provide the information to distinguish fibers with different directions, so it was used to specify the constraint regions. In color FA map, it is clear that one curved fiber - the main segment of cingulum – is arching over the corpus callosum with continuous changed diffusion directions. The regions were placed on the front end of anterior cingulum, the middle of cingulum, and the end of posterior cingulum for every subject. These parameters were used for SLT: step size = 1 voxel, curvature <60° for SLT method, FA threshold of seed region = 0.2. No tracking FA threshold restriction was used for SLT. For ACTSE, the defined regions served as the constraints of extracted cingulum skeleton, and the step size of curve evolution was set to 1 voxel. For real DT-MRI data, *r* is set to 2 voxels to represent the possible maximum radius of cingulum.

Similar to the quantitative evaluation method of smith et al. [11], SD of the location of extracted cingulum skeleton was calculated for ACTSE across scans and subjects as a measure of robustness and repeatability. Two fixed positions were selected: the ROI on anterior cingulum (AC) and another ROI on posterior cingulum (PC).

## Results

### Synthetic data

Extracted skeleton by ACTSE was compared to the center line of tracked fiber bundles by SLT on the simulated synthetic data. Fig. 3 (a) shows the ideal center line of simulated fiber tract overlaid on FA map. The blue line shows the ideal central line of simulated curve tract. Light blue region and yellow region are defined as the starting and ending regions for SLT. These regions also served as the constraints for ACTSE. Fig. 3 (b) and (c) were reconstructed with added noise *sd* = *0.003* (*SNR* = *31.28*) and PVE level *t* = *2*; (d) and (e) were reconstructed with noise *sd* = *0.009* (*SNR* = *10.67*) and *t* = *2*. Fig. 3 (b) and (d) show the fiber tractography results by SLT in red; (c) and (e) show the extracted tract skeleton by ACTSE in green. With increasing noise or PVE, fewer fiber bundles reached the end region compared to the starting region by SLT method and there are obvious tracked deflections at low SNR (Fig. 3 (d)). In contrast, ACTSE led to robust tract skeleton extraction even at high noise situation (Fig. 3 (e)).

**Figure 4:**
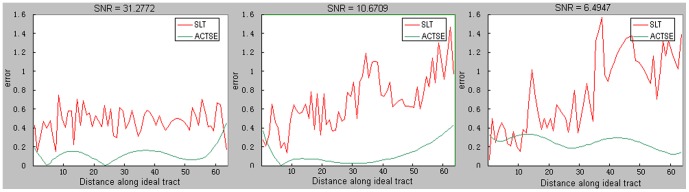
Illustration of deflection errors of ACTSE and SLT along the arc length on simulated phantom. Deflection errors were calculated along the arc length between the ideal tract central line and the skeleton extracted by ACTSE or the center line of tracked fiber tract acquired by SLT on the simulated curve fiber phantom. Red shows the deflection error of calculated center line by SLT method; and green shows the error of extracted skeleton by ACTSE. From left to right, the errors were accessed with filter times *t = 2*, and noise level *sd = 0.003, 0.009, 0.015* respectively.

Fig. 4 plots the deflection error between the tract central line derived by SLT or tract skeleton extracted by ACTSE and the ideal tract central line, at *SNR = 31.28, 10.67, 6.49* corresponding to added noise *sd = 0.003, 0.009, 0.015* respectively at *t = 2*. Table 1 shows the mean error between the skeleton extracted by ACTSE or the center line of tracked fiber tract by SLT and the ideal tract center line at different noise or PVE levels. It is evident that the error of ACTSE is considerably lower than that of SLT under all circumstances investigated.

**Table 1:** Mean error between the skeleton extracted by ACTSE or the center line of tracked fiber tract by SLT and the ideal tract center line at different noise or PVE levels on simulated curve fiber phantom data.

	SLT					ACTSE				
	mean tracking errors									
PVE Level^a^	With random noise SD^b^					With random noise SD^b^				
	0.003	0.006	0.009	0.012	0.015	0.003	0.006	0.009	0.012	0.015
1	0.3998	0.2596	0.3276	0.5298	0.4920	0.0782	0.0908	0.1193	0.3468	0.2134
2	0.4708	0.3639	0.6954	0.4411	0.7839	0.0872	0.1379	0.1161	0.2893	0.2458
3	0.5878	0.6027	0.5672	*NULL*	*NULL*	0.0782	0.3771	0.509	0.7223	0.3654

a The phantom data was filtered by mean filter with window 3×3×3 in variable times (*N* = 1, 2, 3) to simulate different PVE levels.

b Random noise with standard deviation (SD = 0.003,0.006,0.009,0.012,0.015) were added to phantom data to simulate different noise levels.

Along the center line of tracked fiber tract from SLT, the errors were often larger than 1 voxel. For example, with *t* = 2, as shown in Fig. 4(c), some errors from SLT reached 1.5 voxels, while the errors remained lower than 0.4 voxel for ACTSE. With *t* = 3, noise *sd = 0.012 (SNR = 5.56)* or *0.015 (SNR = 4.64)*, no tracked fiber reaching the end region by SLT, while ACTSE was able to identify the skeleton with low error (shown in Table 1). The results proved that ACTSE is robust in identifying the skeleton of simulated curve fiber tract at different noise and PVE levels, showed the superiority for cingulum location by ACTSE compared to tractography methods.

### Experimental DT-MRI data

**Figure 5:**
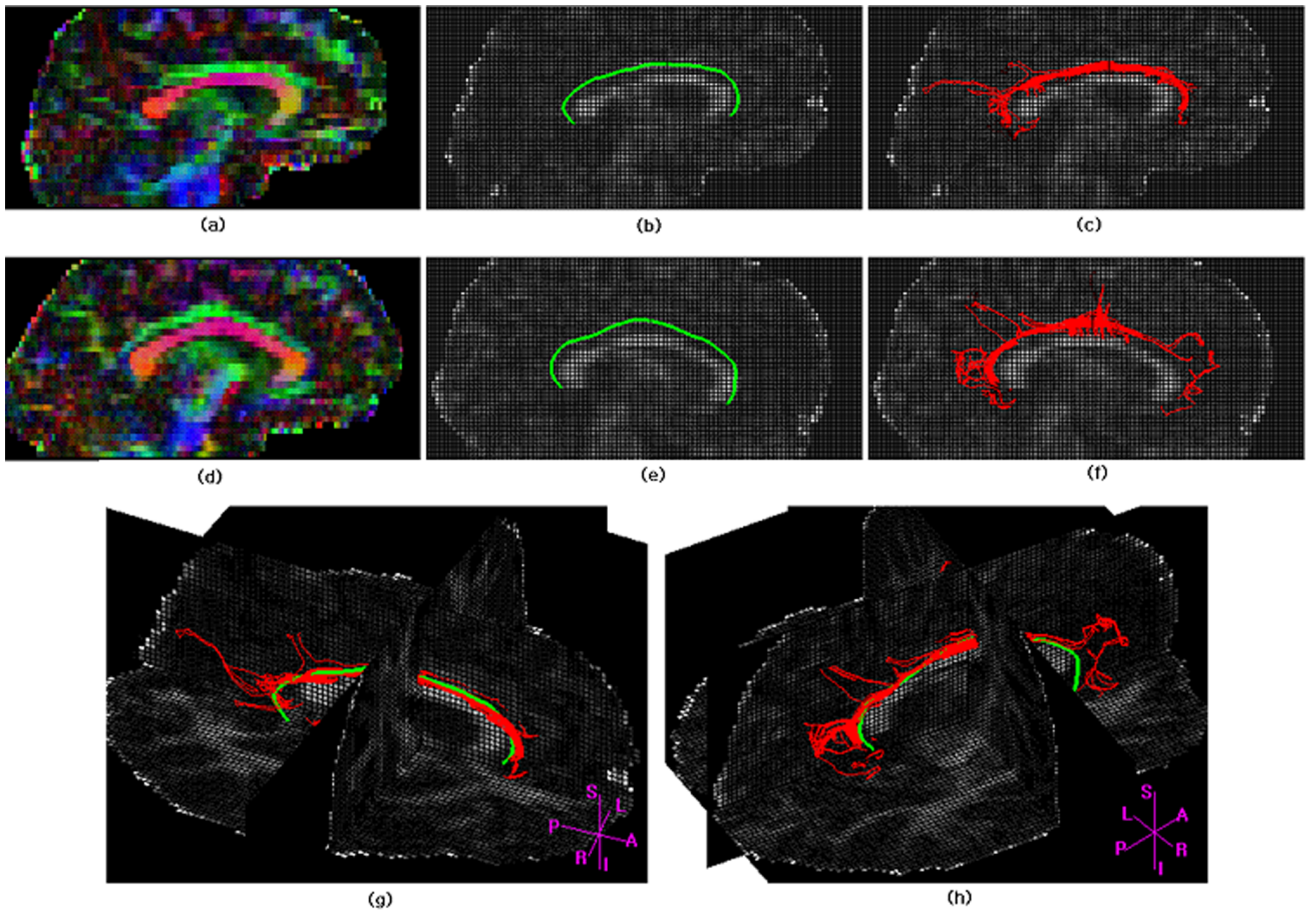
Illustration of skeleton extraction by ACTSE and fiber tracking by SLT for right cingulum. (a), (b), (c) and (g) are from subject 4; (d), (e), (f) and (h) are from subject 6. (a) and (d) show FA color map. (b) and (e) show tract skeleton extraction results of ACTSE (in green) from sagittal view; (c) and (f) show fiber tractography results by SLT (in red) from sagittal view. (g) and (h) show the results of ACTSE and SLT in 3D view.

ACTSE were applied to extract the skeleton of cingulum on 6 healthy subjects. Fig. 5 illustrates the 3D results of skeleton extraction by ACTSE and fiber tracking by SLT on subjects 4 and 6 for right cingulum (from sagittal view and in 3D view). (a), (b), and (c) are from subject 4; (d), (e), and (f) are from subject 6. (b) and (e) show the tract skeleton extraction results by ACTSE (in green) from sagittal view; (c) and (f) show fiber tractography results by SLT (in red) from sagittal view; (g) and (h) show the results of ACTSE and SLT in 3D view. The tract skeleton from ACTSE was consistent with fiber tracking results from SLT, and both results were consistent with fiber directions indicated by color FA maps. However, the fibers derived by SLT also exhibit clearly spurious branches.

**Figure 6:**
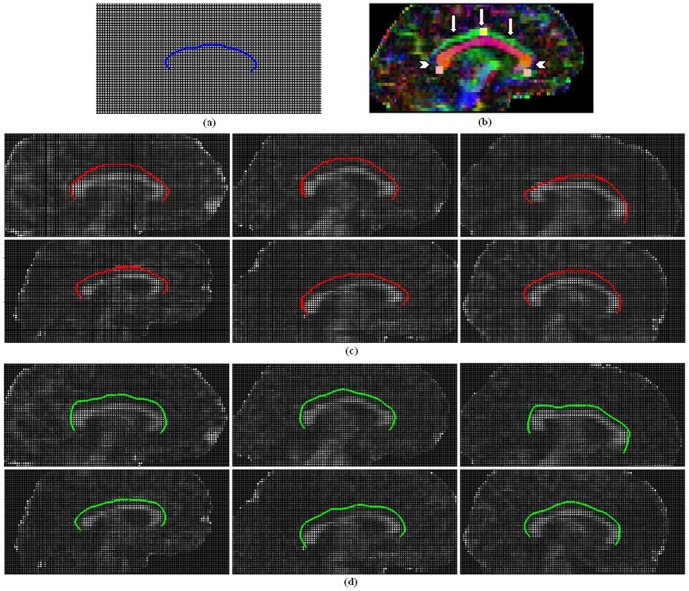
Illustration of 3D skeleton extraction of right cingulum bundle by ACTSE from sagittal view. (a) shows the reference curve of cingulum skeleton (in blue). (b) illustrates the anatomical structure of cingulum on color FA map. Green indicates anterior-posterior; red, left-right; blue, superior-inferior. Cingulum is arching over the corpus callosum. Three ROIs were manually defined on the front end of anterior cingulum, the middle of cingulum, and the end of posterior cingulum for every subject. These regions serve as the constraints of tract skeleton extraction for ACTSE method; for SLT method, these regions also serve as seed ROI (yellow) and end ROIs (pink) for fiber tracking. (c) show the initial curves for skeleton searching by ACTSE of 6 subjects overlaid on sagittal FA maps.. (d) show the extracted cingulum skeletons of 6 subjects overlaid on sagittal FA maps.

Fig. 6. illustrates the 3D tract skeleton extraction of right cingulum bundle by ACTSE on 6 subjects from sagittal view. (a) shows the reference curve of cingulum skeleton. (b) illustrates the anatomical structure of cingulum on color FA map. Three ROIs were manually defined by one expert as the constraint regions for ACTSE; these regions also served as seed ROI and end ROIs for fiber tracking by SLT. (c) show the initial curves for skeleton searching by ACTSE of 6 healthy subjects. (d) show the extracted cingulum skeletons of 6 healthy subjects. These skeletons appeared consistent with the anatomical structure of cingulum.

**Figure 7:**
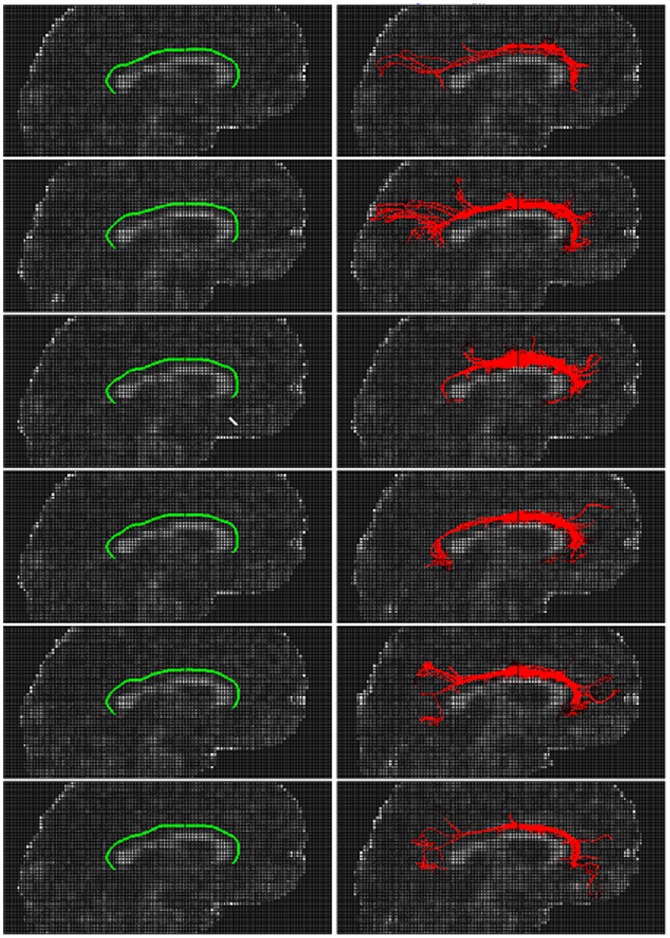
Results illustration of ACTSE and SLT of cingulum at the same noise level from sagittal view. From top to bottom row, they are 6 different volume data with the same noise level. All of them come from the average of random 3 acquisitions of subject 4. Green shows the tract skeleton extraction results by ACTSE (left column) and red shows the fiber tractography results by SLT (right column) correspondingly.

**Figure 8:**
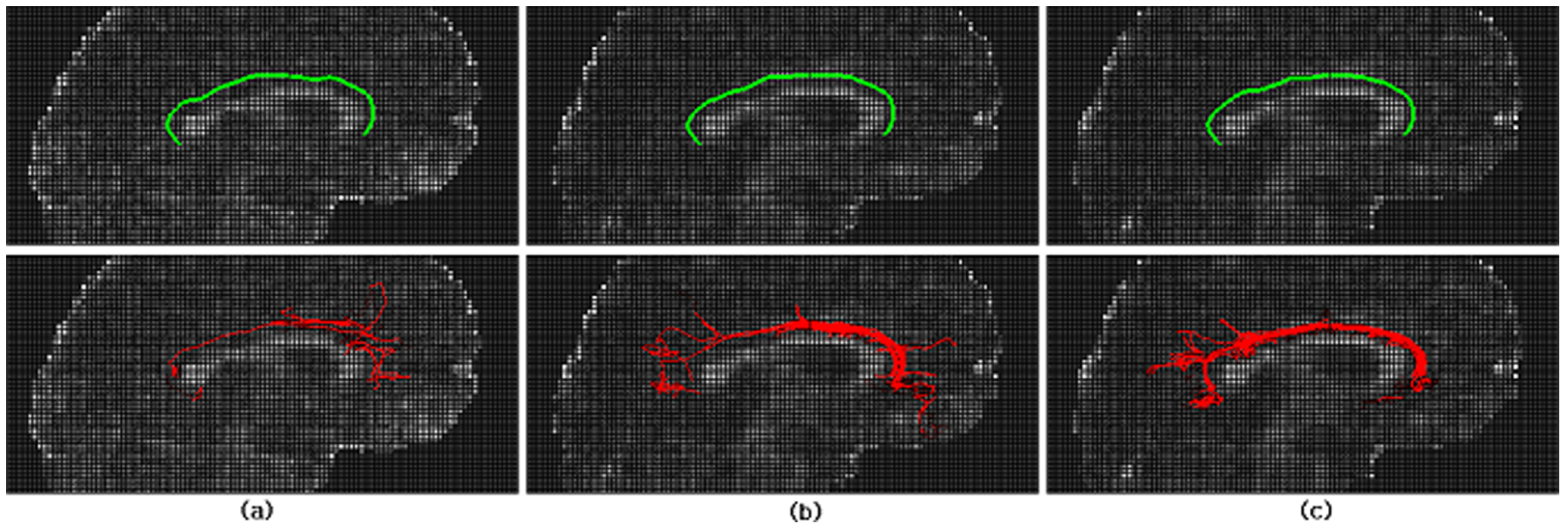
Results illustration of ACTSE and SLT of cingulum at different noise levels from sagittal view. (a), (b) and (c) are the volume data with 3 different noise levels; they were derived from the averages of 1, 3, and 5 acquisitions of subject 4 respectively. From (a), (b) to (c), SNR increased. Green shows tract skeleton extraction results by ACTSE, and red shows the fiber tractography results by SLT correspondingly.

To illustrate the robustness of ACTSE, we showed the cingulum skeleton extraction results of ACTSE at same and different noise levels. To provide a qualitative reference, SLT was also used to show the tracked cingulum. Fig. 7 illustrates the skeleton extraction results by ACTSE and fiber tracking results by SLT of right cingulum on 6 different dataset of subject 4 at the same noise level from sagittal view. All these 6 dataset came from the averages of 3 random acquisitions of subject 4. We can see that the extracted skeleton by ACTSE on the 6 data sets are highly consistent; while tractography results by SLT differ significantly, especially on anterior and/or posterior part of cingulum. Fig. 8 illustrates the skeleton extraction results by ACTSE and fiber tracking results by SLT of right cingulum at different noise levels on subject 4 from saggital view. In Fig. 8, (a), (b) and (c) were derived from data sets of averages from 1, 3, and 5 acquisitions respectively. From (a), (b) to (c), SNR increased. It is evident that ACTSE led to reproducible and reliable results at various SNR levels.

**Figure 9:**
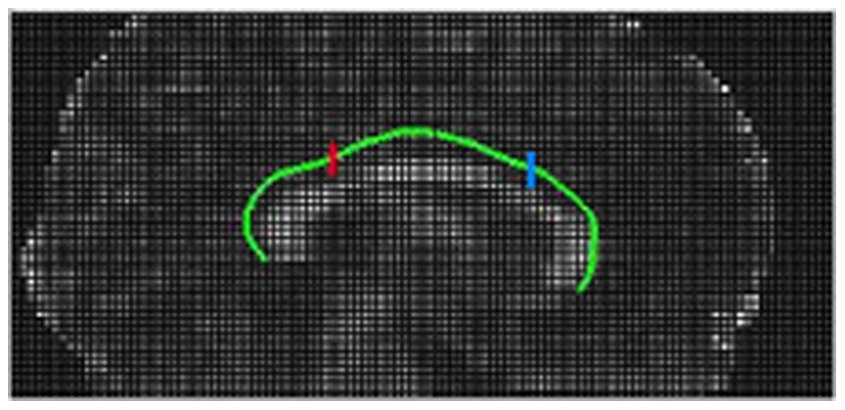
Illustration of locations for SD analysis. Blue and red show the locations on AC and PC for SD analysis respectively.

Two regions with the fixed positions on AC and PC were manually defined by expert for SD analysis for every subject, as illustrated in Fig. 9. As described above, for every subject, the datasets at 3 different noise levels were generated from the average of 5, 3, and 1 acquired scans respectively, and the corresponding noise levels are 1, 2 and 3. From noise level 1 to 3, noise increases and the SNR decreases. SD of the location was estimated on the extracted skeletons by ACTSE at various SNR levels across 6 dataset for every subject. At the position on AC, the average SDs of these six subjects are 0.4313, 0.4396, and 0.5002 voxel for noise level 1, 2, 3 respectively; at another position on PC, the average SDs are 0.2660, 0.3543 and 0.4521 voxel for noise level 1, 2, 3 respectively. Totally with increasing noise, SD increased slightly. At various noise levels, SD is generally lower than 0.67 voxel for every subject, and the average SD of these six subjects is lower than 0.51 voxel. These quantitative analysis results indicated that ACTSE is able to successfully extract the cingulum skeleton under various noise situations and ACTSE is robust and reproducible.

## Discussion

ACTSE was proposed in this paper to extract the tract skeleton of cingulum robustly. This method was evaluated on both synthetic data and experimental DT-MRI data. From Fig. 3 and Table 1, it was proved that ACTSE performed robust at different noise or PVE levels on simulated curved fiber data. From Fig. 5 and 6, it is clear that ACTSE was able to identify the cingulum in various subjects and its results are consistent with known anatomy. From Fig. 7, Fig. 8, and SD analysis, ACTSE showed robustness either at various SNR or on individuals. In general, our proposed ACTSE is robust and reproducible on skeleton extraction of cingulum, which is a very important feature for tract-based clinical/basic analysis.

To provide an optimal trajectory of cingulum, tractography method is usually used. Tractography is to track fiber bundles step by step according to local diffusion tensors, while our ACTSE tries to extract the whole skeleton of cingulum based on active contour model using all the tensors' information throughout the tract simultaneously. For Fig. 3–4 and Fig. 7–8, it is obviously that SLT algorithm is highly sensitive to noise and PVE, and tracking errors will be accumulated along the trajectory, which is consistent with other researcher's results [2], [17], [26]. From Fig. 3 and 4, we can see that with increasing noise or PVE, fewer fiber bundles reached the end region compared to the starting region with SLT method; in contrast, ACTSE led to robust tract skeleton extraction even at high noise situations. From Fig. 7 and 8, we can see that with the SNR changing, the tractography results by SLT differed obviously, especially on anterior and/or posterior part of cingulum; at the high noise situation, SLT even cannot get acceptable tractography results (Fig. 8(a)). While ACTSE performed robustly and consistently in various noise or PVE situations. For the representation of fiber trajectory of cingulum, it seems ACTSE provides a more robust, consistent, and reliable approach.

Smith et al. [11] proposed the method of TBSS, which attempts to combine the strength of voxelwise analyses with the strength of tractography based analyses. TBSS provided the idea of tract skeletonisation to better localize the white matter tracts of the whole brain. It aims to provide the common skeleton of the group subjects, not for individual, although it can provide the skeleton for every subject by “back projection”. During our proposed ACTSE, not only FA but also tensor information is used to provide more accurate localization of cingulum individually. TBSS provide the common skeleton from the group subjects, and registration is very important step. Any error from registration will influence the result of skeleton extraction. Compared to TBSS, ACTSE provides the individual skeleton of cingulum from every subject, and this reduces the requirements of accurate registration. Moreover, in the superior part of the cingulum, TBSS skeletonizes it to a thin surface. In our ACTSE, one consistent line of the skeleton of cingulum was extracted.

Melonakos et al. [37], [38] proposed the Finsler tractography method for white matter connectivity analysis of the cingulum bundle. Although Melonakos et al. adopted active contour model, they used it to construct the direction-dependent cost. Like other tractography method, fiber is tracked by front propagation techniques from seed to target region and then back from target to seed region, which is quite different from ACTSE method. For ACTSE, curve evolution is used for the whole tract searching simultaneously by active contour. Other researchers [39], [40] tried to get the cingulum region by segmentation directly rather than tractography. Edge detection is very important for accurate segmention of cingulum tract. While for ACTSE, the critical issue is to accurately locate the tract pathway of cingulum.

Our results show that ACTSE is robust and it produces smooth and reproducible fiber trajectory of cingulum. The robust performance of ACTSE can be attributed to the use of the active contour model. Active contour model is widely used in computer vision and pattern recognition [33], [34], [35]. It has been proven to be an effective approach to extract object geometric characteristics. In ACTSE, active contour model reduces sensitivity to noise and artifacts in the data and also provides a more flexible framework to incorporate regularization of cingulum skeleton while maintaining consistency with the measured data. Active contour model updates the skeleton based on all the tensors' information throughout the tract simultaneously at each iteration, making it less sensitive to noise and PVE. One requirement for active contour model in ACTSE is the initialization of skeleton contour. If the initial contour is too far from the ideal location, and contour evolution has to pass through more other fiber tracts to reach the ideal location, ACTSE may be failed. To avoid it, we get the initialization by mapping the reference curve to each individual with the constraints of user-defined starting, middle, and end regions. As illustrated in Fig. 6, for these 6 subjects, we got the satisfied skeleton extraction by ACTSE. Moreover, from Fig. 7 and 8, it is proved ACTSE could extract the robust skeleton at various noise levels.

To localize the trajectory of cingulum, like tractography, ACTSE require users to define the start/middle/end regions. For specific analysis of cingulum, both tractography and ACTSE require users' interactions. There are some other methods which provide automatic diffusion analysis of human brain data, such as statistical parametric maps (SPM) and TBSS, which are the popular methods for whole brain diffusion analysis. However, these automatic methods, including SPM and TBSS, aim at the whole brain analysis, so registration is the most important step. The selection of registration method and the selection of filter kernel are still unresolved problems [11], [41]. Any error from registration will led to the error of final analysis results. For more accurate analysis of specific fiber tract, TOI is proposed recently. TOI localizes the trajectory of specific fiber tract individually. However TOI is based on the results of tractography, and any deviation of tractography will lead to inaccurate results for following analysis. Based on the idea of TOI and the tract skeletonisation idea of TBSS, ACTSE provide an approach to localize the skeleton of cingulum accurately and robustly at various noise and PVE situations.

ACTSE was evaluated on real DT-MRI data of six healthy subjects with 12 diffusion directions. 12 diffusion directions is fewer for fiber tracking by tractography methods because of low SNR, and 20 or even 64 diffusion directions were usually acquired. Our study showed that ACTSE could get more robust and reliable cingulum skeleton from DT-MRI data with only 12 diffusion directions, and it is potential to speed up the scan time for data acquisition with fewer diffusion directions for cingulum analysis.

There are widely interests in the study of cingulum [3], [4], [5], [6]. It has been shown that cingulum is involved in many high-level cognitive functions and is an important factor in some psychiatric diseases, such as schizophrenia, depression, Alzheimer's disease, etc. The robust extraction of cingulum skeleton makes it possible for tract-based analysis of cingulum for these clinical psychiatric diseases. For cerebral diseases with space occupying lesions, if part of the cingulum is destroyed or seriously bending, it is not appropriate to extract the whole skeleton of cingulum by ACTSE. The robust localization of cingulum skeleton will result in a robust estimation of diffusion properties along tract skeleton. The evaluation of robustness and reproducibility of skeleton extraction is the most important in our study. How to quantify the diffusion parameters along tract skeleton is another issue. Based on the individual skeleton, similar to TBSS [11], various diffusion parameters, including apparent diffusion coefficient (ADC), FA, and lattice index (LI), can be quantified accordingly along tract skeleton for further analysis. To go back from the skeleton to the whole tract to access the thickness along this fiber tract, directly and simply, we can assess the tract thickness of cingulum by using the FA threshold around the skeleton with some conditions. More other methods could be developed for this in the future. Though ACTSE is proposed for cingulum skeleton extraction, it also provides an idea of skeleton extraction for other tubular-shape fibers.

There are some selective parameters for the model of ACTSE. In our study for the skeleton extraction of cingulum, the weighting parameters *w_a_*, *w_c_*, and *w_fa_* were set to 1; *w_d_* was distinctly set to 0 or 1 according to the calculated angle difference between the contour's tangent vector and the principle eigenvector or the iteration numbers. They worked well for the skeleton extraction of cingulum in our study. The parameters *w_a_* and *w_c_* are the weighting factors related to the anatomical characters of cingulum, *w_d_* could be distinctly set, and *w_fa_* is used to locate the skeleton to the voxels with high FA value. Given the fact that there could be no big difference for the anatomical characters of cingulum and the contrast of FA value between gray matter and white matter for conventional DT-MRI data, it might be feasible to apply these weighting parameters' selection for other DT-MRI data. However, it should be careful that *r* should be selected according to the spatial resolution of DT-MRI data. According to our scanning parameters and the spatial resolution, it was good to set *r* = 2 voxels to represent the possible maximum radius of cingulum. If other scanning parameters are used, *r* should be adjusted and set accordingly. Moreover, although ACTSE has the potential to be used for the skeleton extraction of other tubular fibers, it may be noticed that all the parameters should be checked and adjusted by experiments to make it more efficient.

ACTSE could extract the skeleton of cingulum under various noise situations, which is robust and reproducible. There are some limitations and possible future work. First, although ACTSE is quite convenient and friendly to users' interaction, like tractography methods, this method is not fully automatic and it still requires user's knowledge of anatomy to choose the starting/middle/ending regions. It is possible to “back project” the pre-defined constraint regions from the standard space to every native one, to make the definition of constraint regions more automatic and consistent across subjects. It could be studied in the future. Second, although like tractography, ACTSE provides an approach to robustly extract the orientation and shape of cingulum individually, further studies will be conducted about how to quantify the diffusion parameters along tract skeleton and how to make the comparison across subjects. Third, like other researchers [7], [37]–[40], our study focuses on the main segment of cingulum which arching over corpus callosum. It should be noticed that there is another part of cingulum which parallel to hippocampus. Finally, although ACTSE method has the potential to be extended to the skeleton extraction of other tubular shape fiber tracts, here in our paper ACTSE is proposed specially for cingulum. If apply our approach to other tubular-shape tract, it should be studied further about how to apply these equations and select these parameters. Based on the idea of ACTSE, further studies could be conducted for skeleton extraction of other tracts and to set up the possible anatomic connections between different brain functional regions.

## Conclusion

In summary, we have introduced an approach ACTSE to extract the pathway of cingulum using active contour model based on the tensors' information throughout the tract simultaneously, which allows us to optimize the location of cingulum in a global sense. Validation of this method on synthetic and experimental data proved that ACTSE is able to reduce the influence of noise and PVE, and extract the skeleton of cingulum robustly and reliably. Our proposed method provides an approach to localize cingulum robustly, which is a very important feature for tract-based analysis of cingulum and can be of important practical utility.
